# Activation of Nrf2 Protects against Triptolide-Induced Hepatotoxicity

**DOI:** 10.1371/journal.pone.0100685

**Published:** 2014-07-02

**Authors:** Jia Li, Feihai Shen, Cuiwen Guan, Wenwen Wang, Xiaozhe Sun, Xinlu Fu, Min Huang, Jing Jin, Zhiying Huang

**Affiliations:** 1 School of Pharmaceutical Sciences, Sun Yat-sen University, Guangzhou, China; 2 Pharmaceutical Department, Affiliated Cancer Hospital of Guangzhou Medical University, Guangzhou, China; 3 Center of Laboratory Animals, Sun Yat-sen University, Guangzhou, China; University of South Florida, United States of America

## Abstract

Triptolide, the major active component of *Tripterygium wilfordii* Hook f. (TWHF), has a wide range of pharmacological activities. However, the toxicities of triptolide, particularly the hepatotoxicity, limit its clinical application. The hepatotoxicity of triptolide has not been well characterized yet. The aim of this study was to investigate the role of NF-E2-related factor 2 (Nrf2) in triptolide-induced toxicity and whether activation of Nrf2 could protect against triptolide-induced hepatotoxicity. The results showed that triptolide caused oxidative stress and cell damage in HepG2 cells, and these toxic effects could be aggravated by Nrf2 knockdown or be counteracted by overexpression of Nrf2. Treatment with a typical Nrf2 agonist, sulforaphane (SFN), attenuated triptolide-induced liver dysfunction, structural damage, glutathione depletion and decrease in antioxidant enzymes in BALB/C mice. Moreover, the hepatoprotective effect of SFN on triptolide-induced liver injury was associated with the activation of Nrf2 and its downstream targets. Collectively, these results indicate that Nrf2 activation protects against triptolide-induced hepatotoxicity.

## Introduction

Triptolide, a diterpenoid triepoxide extracted from the Chinese herb *Tripterygium wilfordii* Hook f. (TWHF), is the major active component and the quality control standard of TWHF [1], [2]. Triptolide has exihibited multiple pharmacological activities, such as anti-inflammatory, immune modulation, antiproliferative and proapoptotic activity [3], [4], [5]. The extracts of TWHF containing triptolide have been used for the therapy of inflammation and autoimmune diseases including rheumatoid arthritis, immune complex nephritis and systemic lupus erythematosus [6], [7]. However, it is well known that triptolide has small margin between the therapeutic and toxic doses and could cause serious toxicity on digestive, reproductive, urogenital and blood circulatory systems [8].

Among all the organs, liver is one of the most remarkable targets of triptolide-induced toxicities. In recent years, many studies have shown that various extracts of TWHF containing triptolide could lead to liver injury in animals and humans [9], [10]. An acute toxicity study in mice suggested that liver injury is the main cause of triptolide-induced extraordinary mortality [11]. Furthermore, a pharmacokinetic study in rats has shown that the concentrations of triptolide in liver exceed those observed in other tissues [12]. Although the hepatotoxicity of triptolide has already been reported [13], [14], [15], the implied mechanism has not been fully elucidated yet, and there is still no available strategies in clinic to prevent or alleviate triptolide-induced liver injury.

It was reported in several studies that a possible mechanism for triptolide-induced hepatotoxicity was related to oxidative damage induced by reactive oxygen species (ROS) [13]. Cellular responses to oxidative stress are regulated by a redox-sensitive transcription factor, NF-E2-related factor 2 (Nrf2) [16]. In normal physiological conditions, Nrf2 is anchored in the cytoplasm by Kelch-like ECH-associated protein 1 (Keap1), which also mediates proteasomal degradation of Nrf2. Oxidative and electrophilic stresses cause dissociation of Nrf2 from Keap1 and lead Nrf2 to translocate into the nucleus where it can bind to the Antioxidant Response Element (ARE), a cis-acting element on the promoter of multiple cytoprotective genes. Binding to ARE results in transactivation of these ARE-bearing genes [17], [18]. Typical Nrf2-target genes include xenobiotic-metabolizing enzymes [e.g., glutathione-S-transferases and NAD(P)H:quinine oxidoreductase 1 (NQO1)] and antioxidant-related enzymes [e.g., heme oxygenase-1 (HO-1) and glutamate cysteine ligase (GCL)] [19]. The Nrf2-ARE pathway plays a pivotal role in cellular protection against oxidative damage. Our previous study revealed that Nrf2 played a protective role against triptolide-induced cytotoxicity in rat kidney cells through the counteraction to oxidative stress [20]. However, there is no report currently on the role of Nrf2-ARE defense in triptolide-induced hepatotoxicity.

In the present study, human hepatocellular liver carcinoma cell line (HepG2) was employed to investigate the role of Nrf2 in triptolide-induced hepatotoxicity. A typical Nrf2 agonist (sulforaphane, SFN) was also employed to determine whether activation of Nrf2 could protect against triptolide-induced acute liver injury in BALB/C mice. This study reveals that activation of Nrf2 protects against triptolide-induced hepatotoxicity, and suggests that SFN is a promising agent in protecting livers from triptolide-induced damages.

## Materials and Methods

### Ethics statement

All animal experiments described in this paper have been conducted in strict accordance with the recommendations in the Guide for the Care and Use of Laboratory Animals of Sun Yat-sen University. The protocol was approved by the Animal Ethical and Welfare Committee of Sun Yat-sen University (Approval No: IACUC2012-0902). All procedures were performed under Urethane anesthesia, and all efforts were made to minimize suffering.

### Chemicals and reagents

Triptolide (>99% purity) was purchased from DND Pharm-Technology Co. (Shanghai, China). 3-(4, 5-dimethylthiazol-2-yl)-2, 5-diphenyl-tetrazolium bromide (MTT) was purchased from MP Biomedicals (Santa Ana, CA, USA). Sulforaphane (SFN, ≥98% purity) was purchased from Enzo Life Sciences (Lausen, Switzerland). Anti-NQO1 antibody was purchased from Sigma-Aldrich (St. Louis, MO, USA). Anti-Nrf2 antibody, anti-GCLC antibody and anti-HO-1 antibody were purchased from Santa Cruz Biotechnology (Santa Cruz, CA, USA). Anti-Histone H3 antibody and anti-β-actin antibody were purchased from Cell Signaling Technology (Beverly, MA, USA). Other chemicals were of analytical grade from commercial suppliers.

### Cell culture

HepG2 cells were obtained from the American Type Culture Collection (ATCC, USA) and cultured in Dulbecco's modified Eagle's medium (DMEM, HyClone, Logan, Utah, USA) supplemented with 10% fetal bovine serum (FBS, HyClone, Logan, Utah, USA) and antibiotics (50 U/mL of penicillin and 50 µg/mL streptomycin). The cells were grown in a humidified incubator with 5% CO_2_ at 37°C.

### MTT assay

HepG2 cells were seeded in a 96-well plate at an initial density of 2.5×10^3^ cells/well. Twenty-four hours later, the cells were treated with control (0.1% DMSO) or various concentrations of triptolide for 6, 12, and 24 h. Following treatment, the cells were incubated with 5 mg/mL MTT tetrazolium for 4 h at 37°C. The resulting violet formazan precipitate was solubilized with DMSO (150 µL). After gently shaking at 37°C for 10 min, the absorbance of the dissolved formazan grains within the cells was measured at 492 nm using a microplate reader (Thermo Multskan Ascent 354, USA; Thermo Labsystems, Helsinki, Finland).

### Lactate dehydrogenase (LDH) leakage assay

HepG2 cells were seeded in 24-well plates at a density of 4×10^4^ cells/well and treated with different concentrations of triptolide for 24 h. Cell viability was then assessed by determining the release of LDH from the cells using a LDH Detection Kit (Nanjing Jiancheng Bioengineering Institute, Nanjing, Jiangsu, China) according to the manufacturer's protocol. Enzyme activity was expressed as the percentage of extracellular LDH activity relative to the total LDH activity.

### Determination of intracellular ROS

The fluorescent probe DCFH-DA was used to determine the intracellular accumulation of ROS. Briefly, HepG2 cells were seeded in 96-well black plates at a density of 4×10^3^ cells/well, incubated overnight, and then exposed to triptolide (10, 20, 40, 80 nM) for 6 h. After treatment, the cells were washed with PBS and then stained with DCFH-DA (10 µM) for 30 min at 37°C. The cells were then washed three times with PBS, and the fluorescent intensity of the cells was measured by a Monochromator-Based Multimode Microplate Reader (Infinite M1000, TECAN, Switzerland) at an excitation wavelength of 488 nm and emission wavelength of 525 nm. The raw data from each individual experiment were normalized to vehicle-treated cells.

### Determination of intracellular Glutathione (GSH)

For the measurement of GSH content, HepG2 cells (2.5×10^5^ cells/well) were seeded in 6-well plates and grown overnight. After exposure to triptolide (10, 20, 40, 80 nM) for 6 h, the treatment cell pellets were lysed by ultrasonication. Following centrifugation (4×10^3^ g for 10 min at 4°C), the supernatant (cell extract) was maintained on ice until assayed for the cellular GSH by a GSH Detection Kit (Nanjing Jiancheng Bioengineering institute, Nanjing, Jiangsu, China) according to the manufacturer's instructions.

### Transient transfection with Nrf2 expression plasmid and siRNA

The expression vectors for the dominant positive Nrf2 (PEF-NRF2) and the empty vector (PEF) were kindly provided by Dr. Shinya Ito (The Hospital for Sick Children, Toronto, Canada) [21]. Transient transfection of HepG2 cells was carried out using Lipofectamine 2000 following the manufacturer's instructions. For six-well plates, the HepG2 cells, at approximately 80% confluence, were transfected with 1.5 µg PEF_NRF2 or PEF for 24 h, followed by Western blot analysis to determine the level of Nrf2 over-expression, or treated with triptolide.

Negative control siRNA and siRNA targeting human Nrf2 (5′- GAGAAAGAAUUGCCUGUAAdTdT-3′ and 3′-dTdT CUCUUUCUUAACGGACAUU-5′) were obtained from Ribo (Guangzhou, Guangdong, China). HepG2 cells were transiently transfected with 50 nM of negative control siRNA or Nrf2 siRNA mixed with Lipofectamine 2000 according to the manufacturer's instructions. At 48 h after transfection, the cells were either harvested for Western blot analysis or treated with triptolide.

### Animals

Male BALB/C mice (6 weeks) were perchased from the Laboratory Animals Center of Sun Yat-sen University, Guangzhou, China. All mice were kept in a specific pathogen-free animal facility under controlled conditions at the temperature (24±2°C), humidity (55±15%), with a 12-h light–dark cycle. Food and water were provided ad libitum.

### Experimental protocol

To investigate the protective effect of SFN on triptolide-induced hepatotoxicity, 24 mice were randomly assigned to the following four groups: (1) control group; (2) SFN group (25 mg/kg); (3) triptolide group (1.0 mg/kg); (4) SFN (25 mg/kg) plus triptolide (1.0 mg/kg) group. Compounds were administered through intraperitoneal (i.p.) injection. The mice in the second and fourth groups were injected with SFN 12 h before, 0.5 h before and 12 h after the injection of triptolide. In all treated groups, mice were anesthetized 24 h after triptolide injection and blood samples were collected for serum biochemical assays. Livers were removed and weighed immediately. For histopathological examination, one lobule of the liver was fixed in 10% formalin. The remaining parts of the liver were collected for biochemical analysis.

### Biochemical assays

Serum samples were assayed for alanine transaminase (ALT), aspartate transaminase (AST), alkaline phosphatase (ALP) and LDH by using commercially available enzymatic assay kits (Leadman Group Co., Ltd., Beijing, China).

### Tissue Processing and Staining

The specimens were immersed in a formaldehyde solution for 24 hours. The recipe for formaldehyde solution is 10% of a 37–40% formaldehyde and 90% of a 0.01 mol/L pH 7.4 PBS. After fixation, the specimens were transferred to 70% ethanol and kept there until processed. The specimens were processed through graded alcohols, cleared in Van-clear (substitute for xylene) and embedded in paraffin. Sections of 3 µm were cut and stained with hematoxylin & eosin for overall morphological evaluation.

### Antioxidant enzymes

Activities of superoxide dismutase (SOD), catalase (CAT), glutathione S-transferase (GST) and glutathione peroxidase(GPx) of homogenized liver were measured using commercial kits (Nanjing Jiancheng Bioengineering Institute, Nanjing, Jiangsu, China). GSH was also measured using a GSH Detection Kit (Nanjing Jiancheng Bioengineering institute, Nanjing, Jiangsu, China) according to the manufacturer's instructions.

### Western blot analysis

Cytoplasmic and nuclear extracts were prepared using a Nuclear Extract kit (Active Motif, Carlsbad, CA, USA) according to the manufacturer's recommendations. Equivalent amounts of protein were separated by 12% SDS-polyacrylamide gel electrophoresis and transferred to polyvinylidene difluoride membranes (Millipore Co., Billerica, MA, USA). After being blocked in 5% non-fat milk in TBST [10 mM Tris-HCl (pH 8.0), 150 mM NaCl, 0.1% Tween 20] for 2 h at room temperature, the membranes were incubated with the appropriate primary antibodies at 4°C overnight. The immunoblots were then incubated with a secondary antibody conjugated with horseradish peroxidase for 1 h at room temperature. The membranes were developed using an electrochemiluminescence (ECL) kit (Thermo Scientific/Pierce, Rockford, IL, USA) according to the manufacturer's protocol. The signals were detected by a chemiluminescence detection system (Bio-Rad Laboratories, Hercules, CA, USA). The density of the immunoreactive bands was analyzed using ImageJ 1.41 (National Institutes of Health, Bethesda, Maryland, USA).

### Quantitative real-time PCR analysis

Total RNA was prepared using the RNAiso Plus (TaKaRa, Japan) according to the manufacturer's protocol. The quantity and purity of RNA were assessed by absorbance at 260 and 280 nm. The cDNA was prepared from the total RNA (1 µg) with a reverse transcriptase(RT) Primer Mix using the PrimeScript RT reagent Kit with gDNA Eraser (TaKaRa, Japan) according to the manufacturer's instructions. The primers for real-time PCR analysis were listed in Table 1. The subsequent PCR amplification was carried out on a LightCycler 2.0 system (Roche Diagnostics, Basel, Switzerland) using 40 or 45 cycles of 95°C for 5 s and 60°C for 20 s. β-actin was used as an internal control. Fold changes, expressed as the mean ± standard deviation (SD), were calculated for the treated groups versus the vehicle control using the 2^−ΔΔCT^ method.

**Table 1:** Primer sequences for PCR amplification.

Gene	Species	Type	Sequence
Nrf2	Human	Forward Primer	5′-CGCAGACATTCCCGTTTGTAGA-3′
		Reverse Primer	5′-GTGACCGGGAATATCAGGAACAAG-3′
NQO1	Human	Forward Primer	5′-GTGGCAGTGGCTCCATGTACTC-3′
		Reverse Primer	5′-CTTGGAAGCCACAGAAATGCAG-3′
GCLC	Human	Forward Primer	5′-TTCCTGGACTGATCCCAATTCTG-3′
		Reverse Primer	5′-CTCATCCATCTGGCAACTGTCATTA-3′
HO-1	Human	Forward Primer	5′-TTGCCAGTGCCACCAAGTTC-3′
		Reverse Primer	5′-TCAGCAGCTCCTGCAACTCC-3′
GAPDH	Human	Forward Primer	5′-GCACCGTCAAGGCTGAGAAC-3′
		Reverse Primer	5′-TGGTGAAGACGCCAGTGGA-3′
NQO1	mouse	Forward Primer	5′-CAGCCAATCAGCGTTCGGTA -3′
		Reverse Primer	5′-CTTCATGGCGTAGTTGAATGATGTC -3′
GCLC	mouse	Forward Primer	5′-CAGTCAAGGACCGGCACAAG -3′
		Reverse Primer	5′- CAAGAACATCGCCTCCATTCAG-3′
HO-1	mouse	Forward Primer	5′-TGCAGGTGATGCTGACAGAGG -3′
		Reverse Primer	5′- GGGATGAGCTAGTGCTGATCTGG -3′
GAPDH	mouse	Forward Primer	5′-TGTGTCCGTCGTGGATCTGA -3′
		Reverse Primer	5′-TTGCTGTTGAAGTCGCAGGAG -3′

### Statistical analysis

SPSS v16.0 software (SPSS Inc., Chicago, IL, USA) was used for the statistical analysis. The values were expressed as the mean ± SD. Statistical comparisons were made using Student's *t*-test and one-way analysis of variance (ANOVA), followed by Tukey's test. The level of significance was set at *P*<0.05 or *P*<0.01.

## Results

### Cytotoxicity of triptolide in HepG2 cells

**Figure 1:**
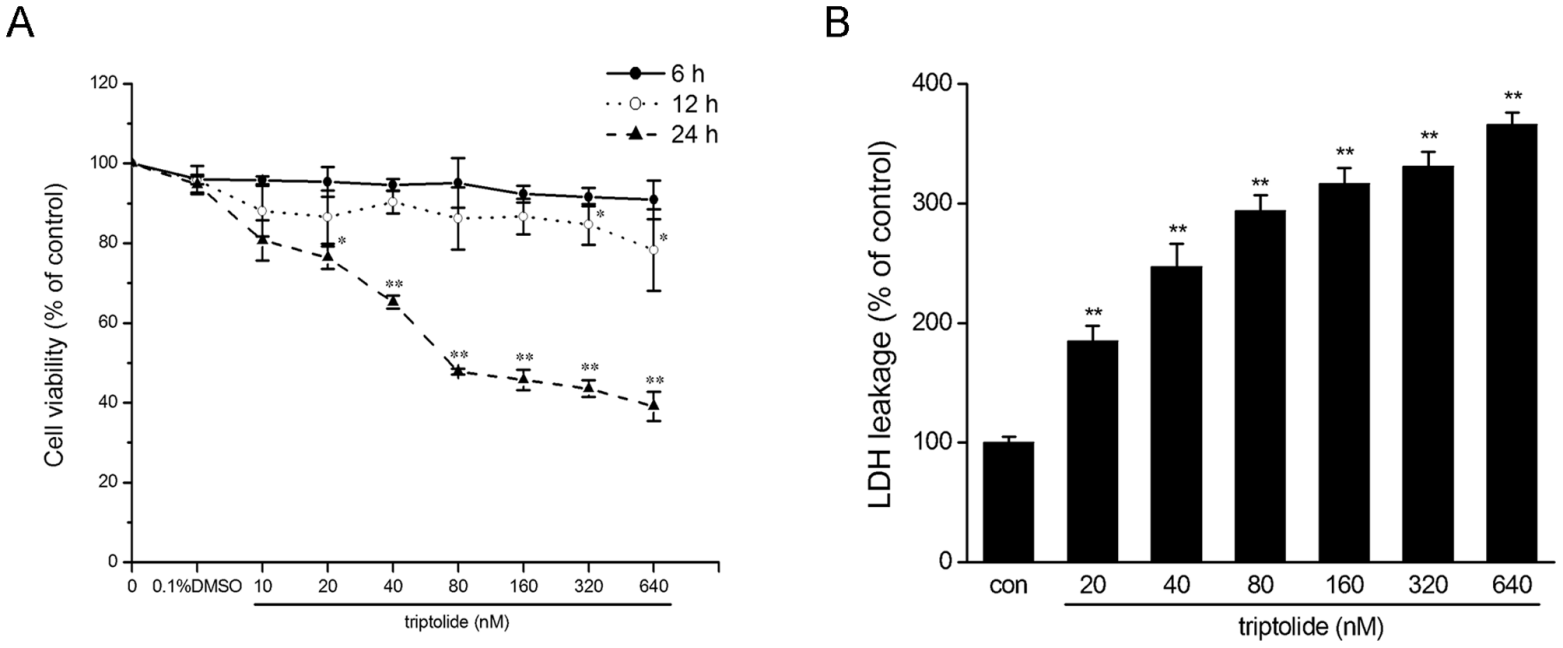
Triptolide induces cytotoxicity in HepG2 cells. (A) HepG2 cells were treated with triptolide at various concentrations for 6, 12 and 24 h. Cell viability was determined by an MTT assay. The viability of the cells without triptolide treatment is defined as 100%. (B) HepG2 cells were treated with triptolide at various concentrations for 24 h. LDH leakage was measured. The data are represented as the mean ± SD from three independent experiments. *P<0.05, **P<0.01 versus vehicle control (0.1% DMSO).

HepG2 cells were employed for investigating the potential toxicity of triptolide on human liver cells *in vitro*. After treatment with 10 to 640 nM of triptolide for 6, 12 and 24 h, cell viability was assessed using the MTT assay. As shown in Fig. 1A, the viability of HepG2 cells decreased in a dose- and time-dependent manner. The IC_50_ determined after 24 h exposure to triptolide was 157.87±2.67 nM.

The cytotoxic effect of triptolide was also demonstrated through measuring the LDH leakage, which is a biomarker indicating the integrity of the cell membrane. 24 h incubation with various concentrations of triptolide (from 20 to 640 nM) markedly induced the release of LDH (Fig. 1B).

### Induction of oxidative stress by triptolide in HepG2 cells

**Figure 2:**
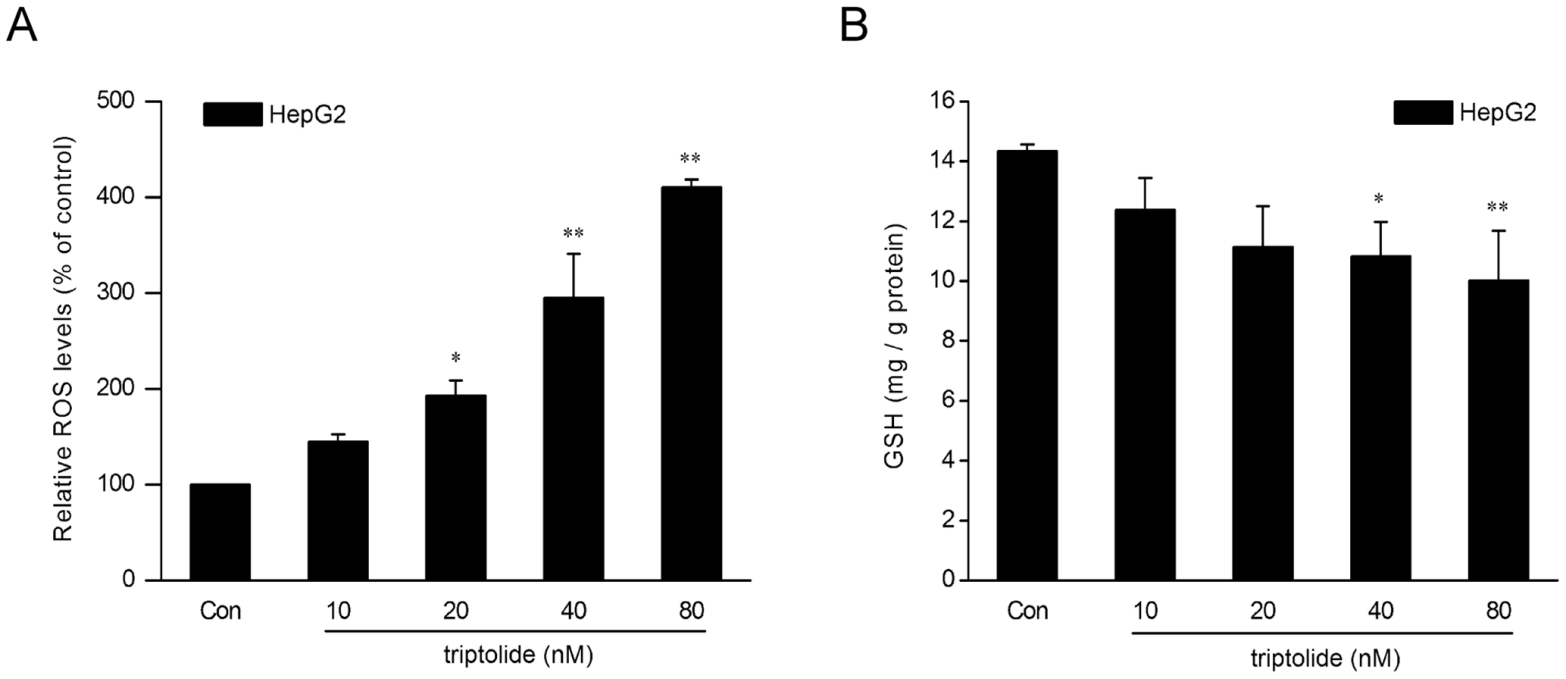
Triptolide induces oxidative stress in HepG2 cells. HepG2 cells were treated with triptolide at various concentrations for 6(A) and GSH (B) were measured respectively using the fluorescent probe DCFH-DA and a GSH Detection Kit. The data are represented as the mean ± SD from three independent experiments. *P<0.05, **P<0.01 versus vehicle control. Con: control (0.1% DMSO).

It has been reported that oxidative damage might be a possible mechanism for triptolide-induced hepatotoxicity [15]. To determine the effects of triptolide on oxidative stress markers involving generation of ROS and intracellular GSH levels, HepG2 cells were exposed to various concentrations of triptolide for 6 h. As shown in Fig. 2A, the intracellular ROS was increased by 44.6, 93.0, 195.0 and 310.3% at 10, 20, 40 and 80 nM triptolide, respectively. Triptolide significantly reduced the intracellular levels of GSH in a dose-dependent manner, with 80 nM triptolide resulting in the most severe depletion (Fig. 2B). These results indicated that triptolide could induce oxidative stress in HepG2 cells.

### Protection against triptolide-induced cytotoxicity by Nrf2 in HepG2 cells

**Figure 3:**
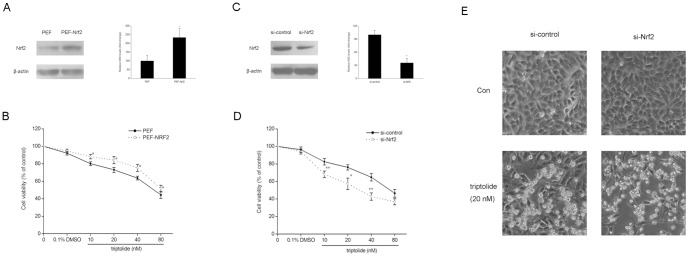
The role of Nrf2 in the protection against triptolide-induced cytotoxicity in HepG2 cells. Overexpression experiments (A, B): HepG2 cells were transiently transfected with PEF or PEF_NRF2 plasmids. The cells were treated 24 h later with triptolide at various concentrations for 24 h. (A) Western blot analysis of Nrf2 expression 24 h after transfection. Expression of β-actin was used as an internal control. Representative blots from three independent experiments are shown. The density of the immunoreactive bands was analyzed, and the data are represented as the means ± SD. (B) Cytotoxicity was determined by an MTT assay. The data are represented as the mean ± SD from three independent experiments. Knockdown experiments (C, D, E): HepG2 cells were transiently transfected with control siRNA or siRNA targeting Nrf2. The cells were treated 48 h later with triptolide at various concentrations for 24 h. (C) Western blot analysis of Nrf2 expression 48 h after transfection. Expression of β-actin was used as an internal control. A representative blot from three independent experiments is shown. The density of the immunoreactive bands was analyzed, and the data are represented as the means ± SD. (D) Cytotoxicity was determined by an MTT assay. The data are represented as the mean ± SD from three independent experiments. (E) Morphological changes in normal and Nrf2 knockdown HepG2 cells treated with triptolide (20 nM) were observed under an inverted phase contrast microscope (100×, Olympus, Japan). The representative results from three independent experiments are shown. *P<0.05, **P<0.01 versus control cells treated with the same concentration of triptolide.

Since Nrf2 is regarded as one of the most important regulators in the cell to counteract oxidative stress [16], the role of Nrf2 in triptolide-induced cytotoxicity was evaluated in an Nrf2 overexpression model to find out whether Nrf2 could attenuate triptolide-induced cytotoxicity. HepG2 cells were transiently transfected with either Nrf2 expressing plasmid or scrambled plasmid, treated with various concentrations of triptolide (from 10 to 80 nM) for 24 h and subjected to MTT analysis. The efficiency of Nrf2 overexpression at the protein level was assayed by western blotting (Fig. 3A). As shown in Fig. 3B, Nrf2 significantly attenuated triptolide-induced cytotoxicity in HepG2 cells, which suggested a protective role of Nrf2 in triptolide-induced hepatotoxicity.

To confirm the cellular protection of Nrf2 against triptolide-induced hepatotoxicity, siRNA was used to silence Nrf2 in HepG2 cells.As shown in Fig. 3C, siRNA-Nrf2 transfection led to a knockdown of Nrf2 protein as determined by Western blot analysis. Nrf2 knockdown significantly increased triptolide-induced viability reduction in HepG2 cells (Fig. 3D). Morphological analysis also revealed a more severe toxic effect of triptolide in Nrf2 knockdown cells compared with the control cells (Fig. 3E). These combined results indicate that Nrf2 knockdown is correlated with high susceptibility to triptolide-induced toxicity in HepG2 cells.

**Figure 4:**
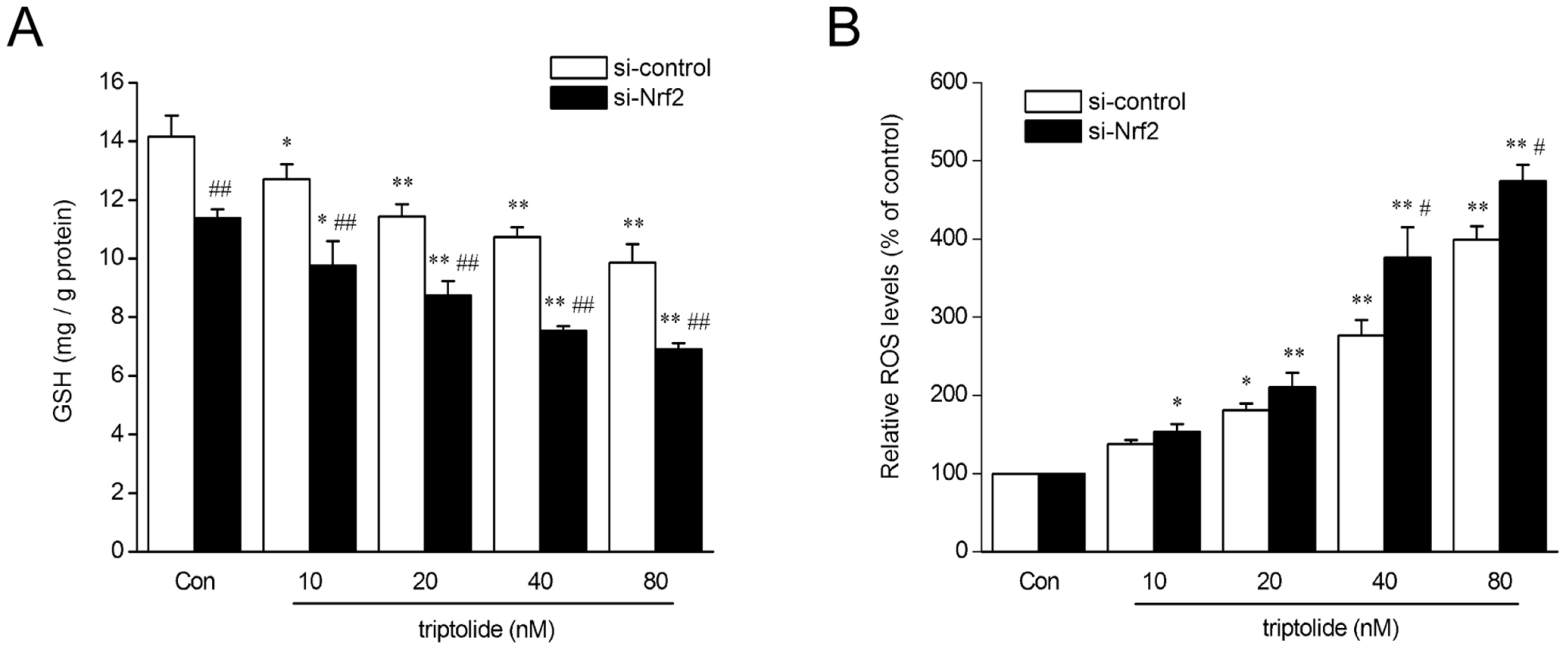
siRNA-Nrf2 enhances triptolide-induced oxidative stress in HepG2 cells. HepG2 cells transfected with siRNA-control or siRNA-Nrf2 were treated with triptolide at various concentrations for 6 h. (A) Levels of intracellular GSH were measured. (B) The intracellular ROS levels were measured using the fluorescent probe DCFH-DA.The data are represented as the mean ± SD from three independent experiments. *P<0.05, **P<0.01 versus vehicle control; ^#^ *P*<0.05, ^##^ *P*<0.01 versus si-control cells treated with the same concentration of triptolide. Con: control (0.1% DMSO).

To further explore whether the enhanced cytotoxicity of triptolide induced by Nrf2 knockdown was relative to oxidative stress, intracellular levels of GSH and ROS after triptolide (from 10 to 80 nM) treatment were determinded in both Nrf2 knockdown cells and control cells. As shown in Fig. 4A, after triptolide treatment, intracellular GSH decreased dose-dependently in both type of cells, and were at lower levels in Nrf2 knockdown cells compared with that of control cells treated with the same concentration of tripotlide. Nrf2 knockdown cells produced higher levels of ROS than control cells, and both of them displayed a dose-dependent manner (Fig. 4B). These results indicated that the enhanced cytotoxicity of triptolide produced by Nrf2 knockdown is possiblely due to the reduced capacity of HepG2 cells against oxidative stress.

### Protective effect of SFN on triptolide-induced liver injury in BALB/C mice

To verify the protective effect of Nrf2 activation on triptolide-induced hepatotoxicity *in vivo*, and to seek a potential theraputic strategy, we established an animal model of acute liver injure induced by triptolide and investigated the effect of SFN, a typical Nrf2 agonist, on triptolide-induced liver injury.

As shown in Table 2, in the triptolide-treated group, serum activities of AST, ALT and LDH increased 9.1-, 9.8- and 3.0- fold, respectively (*P<0.01*). In contrast, administration of SFN in the triptolide-treated group significantly reduced triptolide-induced increase of serum AST, ALT and LDH activities (*P<0.01*). Treatment with SFN alone did not affect the activities of AST, ALT and LDH.

**Table 2:** Effect of triptolide and SFN on ALT, AST, ALP and LDH in BALB/C mice.

Parameters	Groups			
	Con	SFN	TP	SFN+TP
**AST (U/L)**	154.7±32.3	170.2±36.9	1404.3±457.0^b^	171.2±41.2^c^
**ALT (U/L)**	45.7±6.3	65.9±22.5	447.2±105.9^b^	97.2±29.1^a^^,^ c
**ALP (U/L)**	263.2±24.9	232.6±33.8	184.5±29.2	183.2±30.1
**LDH (U/L)**	1455.3±103.0	1699.0±365.2	4395.7±286.5^b^	1221.3±130.6^c^

Mice were given vehicle control, SFN (25 mg/kg), TP (1.0 mg/kg), SFN (25 mg/kg)+TP (1.0 mg/kg) by intraperitoneal injection. Mice were sacrificed under anesthesia 24 h after triptolide treatment. Values are expressed as mean ± SD. n = 6.

a *P<0.05* compared to the control group.

b *P<0.01* compared to the control group.

c *P<0.01* compared to the triptolide-treated group.

**Figure 5:**
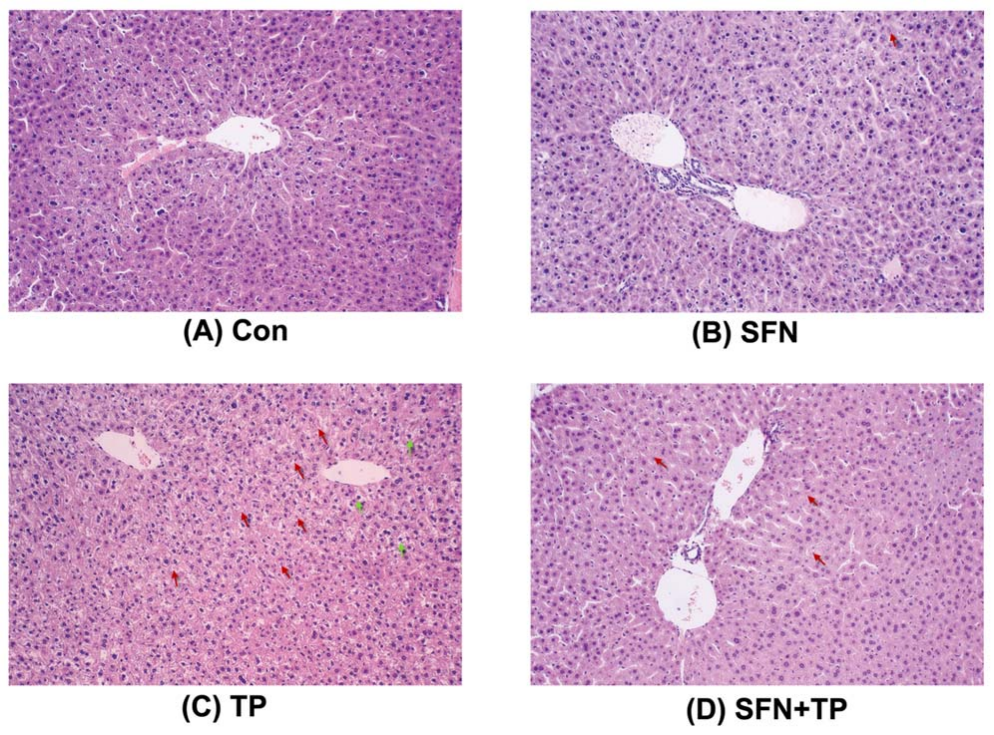
Photomicrographs of hematoxylin and eosin-stained liver sections. Mice were sacrificed 24-µm intervals and then stained with hematoxylin and eosin for light microscopic analysis (×100). The photomicrographs are of livers obtained on (A) Con group, (B) SFN group, (C) TP group, (D) SFN+TP group. Green arrows indicated hepatocellular hydropic degeneration, red arrows indicated necrosis.

Histopathological analysis of livers showed severe hepatocellular hydropic degeneration (green arrows) and necrosis (red arrows) which occurred in triptolide alone treated mice but not in control animals. The levels of hepatocellular hydropic degeneration and necrosis were significantly decreased in the SFN plus triptolide treated group (Fig. 5). Taken together, these results indicate that SFN is able to prevent triptolide-induced liver injury in mouse.

### Protective effect of SFN on triptolide-induced oxidative stress in BALB/C mice liver

Oxidative stress was quantified through measuring enzyme activities of SOD, CAT, GST, GPx and levels of GSH in liver tissue homogenates. Significant reduction in the activities of SOD (*P<0.05*), CAT (*P<0.01*) and GST (*P<0.01*) were found in triptolide-treated group when compared with those of the control group. SFN treatment resulted in a significant increase in the activities of SOD, CAT and GST. GPx activity was also decreased in triptolide-treated mice, but there was no statistically significant difference (*P>0.05*). Concentrations of GSH in the liver were significantly lower with triptolide treatment compared with control (*P<0.01*). This decrease was significantly increased by SFN treatment (*P<0.01*) (Table 3).

**Table 3:** Effect of triptolide and SFN on activities of antioxidant enzymes in liver of BALB/C mice.

Parameters	Groups			
	Con	SFN	TP	SFN+TP
**SOD (U/mg prot)**	18.7±0.8	19.4±0.8	8.8±3.2^b^	13.8±0.9^b^^,^ d
**CAT (U/mg prot)**	72.9±5.9	64.7±16.7	38.4±6.8^a^	66.5±17.7^c^
**GST (U/mg prot)**	184.0±11.8	197.8±12.9	157.2±4.0^a^	194.3±3.4^d^
**GPx (U/mg prot)**	1429.8±76.3	1532.3±178.0	1250.7±53.8	1465.2±122.2
**GSH (mg/g prot)**	28.8±1.9	30.9±1.1	19.2±0.4^b^	30.6±0.6^d^

Mice were given vehicle control, SFN (25 mg/kg), TP (1.0 mg/kg), SFN (25 mg/kg)+TP (1.0 mg/kg) by intraperitoneal injection. Mice were sacrificed under anesthesia 24 h after triptolide treatment. Values are expressed as mean ± SD. n = 6.

a *P<0.05* compared to the control group.

b *P<0.01* compared to the control group.

c *P<0.05* compared to the triptolide-treated group.

d *P<0.01* compared to the triptolide-treated group.

### Effect of SFN on Nrf2-ARE activation in BALB/C mice liver

**Figure 6:**
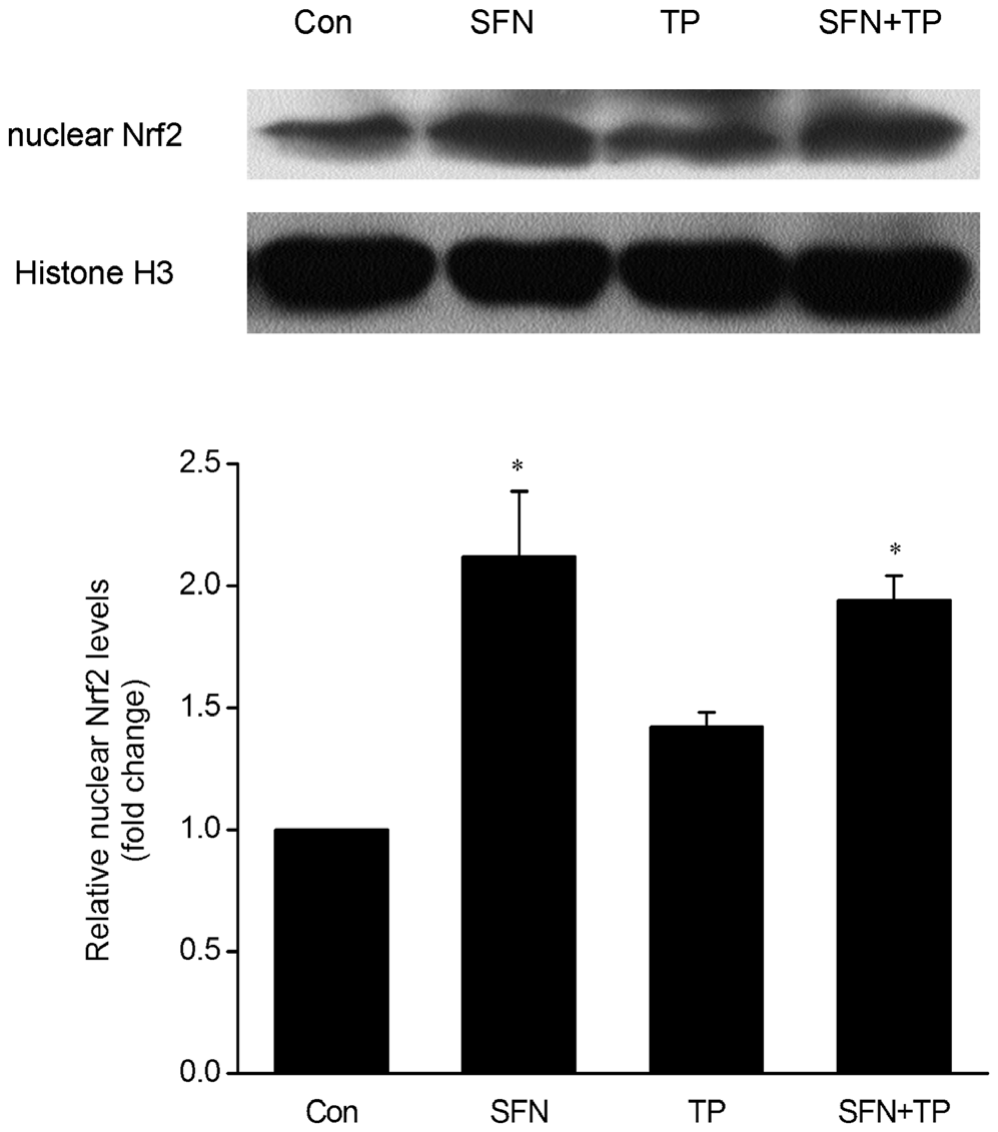
Effect of triptolide and SFN on nuclear levels of Nrf2 in livers of BALB/C mice. Nuclear protein lysates were made from livers of mice from different experimental groups and subjected to Western blot analysis using the indicated antibodies. The histone H3 was used as an internal control. A representative blot from three independent experiments is shown. The density of the immunoreactive bands was analyzed, and the data are represented as the means ± SD; n = 6; *P<0.05 versus Con group.

To explore the potential mechanisms implied in the protective effect of SFN on triptolide-induced liver injury, we determined the nuclear accumulation of Nrf2 by Western blot. Fig. 6 showed that the accumulation of Nrf2 in the nuclear fraction was slightly higher in the livers of triptolide-treated mice than that of the control mice (*P>0.05*). The Nrf2 accumulations in the liver nuclear fraction were significantly increased in SFN-treated group and in the combined treatment group, as compared with vehicle control group (*P<0.05*).

**Figure 7:**
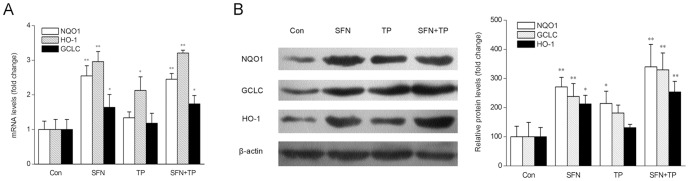
Effects of triptolide and SFN on Nrf2-related downstream targets in livers of BALB/C mice. (A) The mRNA levels of NQO1, GCLC and HO-1 were analyzed by real-time PCR. GAPDH was used as an internal control. The data are represented as the mean ± SD; n = 6; *P<0.05, **P<0.01 vs Con group. (B) The protein levels of NQO1, GCLC and HO-1 were analyzed by Western blot. β-actin was used as an internal control. A representative blot from three independent experiments is shown. The density of the immunoreactive bands was analyzed, and the data are represented as the means ± SD; n = 6; *P<0.05, **P<0.01 versus Con group.

To confirm the effects of SFN on Nrf2-related downstream targets, we analyzed liver tissues by Q-PCR and Western blot. Fig. 7A showed that SFN treatment caused dramatic increases in NQO1 (*P<0.01*), HO-1 (*P<0.01*) and GCLC (*P<0.05*) mRNA levels. Moreover, the protein levels of NQO1, HO-1 and GCLC were significantly increased in SFN treated group and in the combined treatment group (Fig. 7B). These results indicate that Nrf2 activation is involved in the protective effect of SFN on triptolide-induced liver injury.

## Discussion

Triptolide, one of main active components in Chinese medicinal herb TWHF, has exihibited various pharmacological activities. However, multiple organ toxicity of triptolide, especially its hepatotoxicity, limits its clinical application to a great extent. Until recently, the hepatotoxicity of triptolide has not been well characterized. In this study, we found that triptolide caused oxidative stress and cell damage in HepG2 cells, and revealed the protective role of Nrf2 in triptolide-induced cytotoxicity. Furthermore, we provided evidence with an animal model confirming the protective effect of Nrf2 activation against triptolide-induced hepatotoxicity. The results showed that triptolide-induced liver injury was significantly alleviated after administration of SFN, a typical Nrf2 agonist, which suggested that SFN could be used as a promising agent in protecting livers from triptolide-induced damages.

The present study focused on oxidative stress, which has been implicated in the hepatotoxicity of triptolide [13]. Nrf2 is a cellular sensor of chemical-induced oxidative stress and functions to restore homeostasis by up-regulating antioxidant, xenobiotic-metabolizing, and other cytoprotective enzymes [22]. The role of Nrf2 in the liver has been reported, as livers of Nrf2-null mice are more susceptible to various chemical-and oxidative stress-induced pathologies than wild-type mice. Pharmacological activation of Nrf2 by the use of small molecules, such as SFN, oltipraz and the natural triterpenoid oleanolic acid, results in hepatoprotection. For example, numerous studies have revealed that treatment with these Nrf2 activators could decrease liver injury from acetaminophen, carbon tetrachloride, and cadmium [23]. Our previous study indicated that in a rat kidney cell line NRK-52E, Nrf2 was adaptively activated to protect NRK-52E cells from triptolide-induced toxicity by the counteraction against oxidative damage [20]. In this study, the similar results were observed in HepG2 cells (Figures S1, S2, S3). Besides, triptolide-induced cytotoxicity in HepG2 cells was enhanced when Nrf2 was knocked down by siRNA but was reduced when Nrf2 was overexpressed (Fig. 3–5). These results revealed the protective role of Nrf2 in counteraction against triptolide-induced hepatotoxicity. Moreover, we also found that HepG2 cells were more susceptible to triptolide-induced cytotoxicity than NRK-52E cells, as indicated by a lower IC_50_ value and more severe oxidative stress status in HepG2 cells.

We used an animal model to confirm the protective effect of SFN, an Nrf2 agonist, against triptolide-induced hepatotoxicity in further research. SFN is a natural dietary isothiocyanate produced by the enzymatic action of the myrosinase on glucopharanin, a 4-methylsulfinylbutyl glucosinolate contained in cruciferous vegetables of the genus Brassica such as broccoli, brussel sprouts, and cabbage [24]. It shows cytoprotective properties in several *in vivo* experimental models associated with oxidative stress such as carbon tetrachloride-induced hepatotoxicity [25], cisplatin induced-nephrotoxicity [26], and cardiac ischemia and reperfusion [27].

Liver function was assessed by measuring the activities of serum biomarker enzymes (i.e. ALT, AST, ALP and LDH). The increased levels of ALT, AST and ALP are conventional indicators of hepatocellular necrosis [28]. LDH is also an indicator of liver injury, although it has different isoenzymes and is not liver-specific [29]. In the present study, triptolide-induced hepatotoxicity, reflected by increased ALT, AST and LDH activities was significantly alleviated by treatment with SFN. The failure of triptolide in inducing ALP might be because that ALP is a delayed indicator and hasn't responsed in our acute liver injury model [30]. Histopathology analysis of mice liver tissues also showed much reduced areas of hemorrhaging, necrosis, and more normal tissue structures in mice co-treated with SFN compared with the group treated only with triptolide, which is in consistent with the protective role of SFN against triptolide-induced cell morphological changes.

Consistent with previous reports [15], triptolide exposure led to oxidative stress in livers of BALB/C mice as observed by decreased concentrations of GSH and decreased activities of antioxidant enzymes (SOD, CAT and GST). GSH is an endogenous antioxidant, which prevents damage to the cellular components by ROS and peroxides. SOD is an antioxidant enzyme involved in the scavenging of superoxide radicals; it is crucial to maintain the balance between ROS and antioxidant enzymes, which serves as a major mechanism in preventing damage elicited by oxidative stress. CAT protects the cells from oxygen-free radical damage by converting hydrogen peroxide formed by the detoxification of superoxide radicals to molecular oxygen and water before it can decompose to form the highly reactive hydroxyl radical [31]. GST catalyzes the conjugation of electrophiles generated by toxins to GSH via a sulfhydryl group; this activity of GST helps in accelerated metabolism and excretion of the electrophilic molecules. GST might also bind to the toxins and function as transport proteins. GPx is another crucial enzyme that plays an important role in protection against hydrogen and lipid peroxides; the decline in GPx activity is correlated with the increased level of ROS. Our results showed that SFN could remarkably increase the concentration of GSH and enhance activities of SOD, CAT and GST in triptolide-treated mice, and thus protected the liver from triptolide-induced injury. Although there was no significant difference in GPx activities between each group, a similar trend could still be observed (Table 3).

SFN is considered as an indirect antioxidant, and it is able to induce many cytoprotective proteins, including antioxidant enzymes, through activating Nrf2 [24]. The results of the present study showed that SFN treatment significantly enhanced nuclear translocation of Nrf2 and expression of subsequent enzymes, such as NQO1, HO-1 and GCLC. According to other studies, the mechanism for SFN action on Nrf2 might be due to its interaction with Keap1 thiols. It is worthy to notice that nuclear accumulation of Nrf2 and NQO1, HO-1, GCLC expression in the triptolide-induced liver injury group were also slightly higher than those of the controls. This might be due to Nrf2 activation acting as a cellular adaptive response against triptolide-induced toxicity. Nrf2 activation was initiated as soon as the subjects were challenged by triptolide-induced oxidative stress. However, it was unable to completely overcome the toxicity while the adaptively stimulated Nrf2 might alleviate or delay the deleterious effects of triptolide. This possible mechanism is in accordance with other reports [32], [33].

We have demonstrated that activation of Nrf2 by its agonist SFN could protect against triptolide-induced hepatotoxicity *in vitro* and *in vivo*, but since SFN also has other biological targets besides Nrf2, we couldn't exclude other mechanisms implied in the protective effect of SFN. Further studies on the protective activity of SFN on triptolide induced-toxicity are still needed.

In summary, treatment of HepG2 cells with triptolide induced oxidative stress, and Nrf2 acted as a defensive response against the triptolide-induced cytotoxicity. Nrf2 activation *in vivo* by SFN also mitigated triptolide-induced hepatotoxicity. This study provides new insights into the development of strategies to prevent or alleviate triptolide-induced hepatotoxicity.

## Supporting Information

Figure S1 **Effects of triptolide on ARE-dependent transactivation by Nrf2.** (A) HepG2 cells were transfected with a pGL3 plasmid containing the ARE-motif (from the NQO1 promoter). Eighteen hours after transfection, the cells were treated with triptolide (5, 10, 20 nM) for 6 h. (B) HepG2 cells were cotransfected with a PEF empty or a Nrf2 expression vector, and a pGL3-ARE plasmid. Eighteen hours after transfection, the cells were treated with triptolide (20 nM) for 6 h. Luciferase activity was determined by calculating the firefly luciferase to renilla luciferase ratio. Data are expressed as fold of induction of luciferase activity compared to vehicle control (mean ± SD; n = 3). **P<0.01 versus vehicle control. ## *P*<0.01 versus cells transfected with PEF and treated with the same drug. Con: control (0.1% DMSO).
(TIF)

Figure S2 **Effect of triptolide on the nuclear levels of Nrf2 protein in HepG2 cells.** After being exposed to triptolide (20 nM) for the indicated time periods (A) or to the indicated concentrations of triptolide (10, 20, 40, 80 nM) for 6 h (B), the nuclear protein lysates were prepared and subjected to Western blot analysis using the indicated antibodies. The histone H3 was used as an internal control. For a positive control, HepG2 cells were treated with 5 µM sulforaphane (SFN) for 24 h (A) or 6 h (B). A representative blot from three independent experiments is shown. The density of the immunoreactive bands was analyzed, and the data are represented as the means ± SD from three independent experiments. **P<0.01 versus time 0 or vehicle control. Con: control (0.1% DMSO).
(TIF)

Figure S3 **Effects of triptolide on the levels of Nrf2 target genes in HepG2 cells.** HepG2 cells were treated with triptolide (20 nM) for the indicated time periods. The mRNA expressions of NQO1 (A), GCLC (B) and HO-1 (C) were analyzed by real-time PCR. β-actin was used as an internal control. For a positive control, HepG2 cells were treated with 5 µM sulforaphane (SFN) for 24 h. The data are represented as the mean ± SD from three independent experiments. *P<0.05, **P<0.01 versus time 0.
(TIF)

Checklist S1 **ARRIVE Checklist.**
(PDF)

## References

[1] Kupchan SM, Court WA, Dailey RG, Gilmore CJ, Bryan RF (1972) Triptolide and tripdiolide, novel antileukemic diterpenoid triepoxides from Tripterygium wilfordii. J Am Chem Soc 94: 7194–7195. 5072337 10.1021/ja00775a078

[2] Qiu D, Kao PN (2003) Immunosuppressive and anti-inflammatory mechanisms of triptolide, the principal active diterpenoid from the Chinese medicinal herb Tripterygium wilfordii Hook. f. Drugs R D 4: 1–18. 12568630 10.2165/00126839-200304010-00001

[3] Zheng YL, Lin JF, Lin CC, Xu Y (1994) Anti-inflammatory effect of triptolide. Acta pharmacol Sin 15: 540–543. 7709756

[4] Shamon LA, Pezzuto JM, Graves JM, Mehta RR, Wangcharoentrakul S, et al (1997) Evaluation of the mutagenic, cytotoxic, and antitumor potential of triptolide, a highly oxygenated diterpene isolated from Tripterygium wilfordii. Cancer lett 112: 113–117. 9029176 10.1016/S0304-3835(96)04554-5

[5] Lu H, Hachida M, Enosawa S, Li XK, Suzuki S, et al (1999) Immunosuppressive effect of triptolide in vitro. Transplant Proc 31: 2056–2057. 10455969 10.1016/s0041-1345(99)00262-6

[6] Guo JL, Yuan SX, Wang XC, Xu SX, Li DD (1981) Tripterygium wilfordii Hook f in rheumatoid arthritis and ankylosing spondylitis. Preliminary report. Chin Med J 94: 405–412. 6796344

[7] Li LS (1982) Clinical and experimental studies on the effect of Tripterygium wilfordii Hook in the treatment of nephritis. Zhonghua yi xue za zhi 62: 581–585. 6817881

[8] Luo YW, Shi C, Yuan Y, Zhang M, Liao MY (2009) Research progress on the toxicity of triptolide. J Toxicol 23: 74–77.

[9] Xiu JW, Li PX, Min W (2007) Hepatotoxicity Caused by Commonly used Chinese Medicinal Herbs and Compound Preparation. Journal of Capital Medical University 28: 220–224.

[10] Zhi C, Wen JZ, Li G, Yong HW, Hui JF, et al (2011) Experimental Progress of Liver Toxicity of Tripterygium wilfordii and Its Mechanism. Chinese Journal of Experimental Traditional Medical Formulae 17: 243–246.

[11] Hong D, Jian YW, Jing T, Xian FY, Jun C, et al (2004) Acute toxicity of triptolide and the mechanisms involved. Chinese herbal medicine 27: 115–118.

[12] Shao F, Wang G, Xie H, Zhu X, Sun J, et al (2007) Pharmacokinetic study of triptolide, a constituent of immunosuppressive chinese herb medicine, in rats. Biol Pharm Bull 30: 702–707. 17409506 10.1248/bpb.30.702

[13] Mei Z, Li X, Wu Q, Hu S, Yang X (2005) The research on the anti-inflammatory activity and hepatotoxicity of triptolide-loaded solid lipid nanoparticle. Pharmacol Res 51: 345–351. 15683748 10.1016/j.phrs.2004.10.007

[14] Yao J, Jiang Z, Duan W, Huang J, Zhang L, et al (2008) Involvement of mitochondrial pathway in triptolide-induced cytotoxicity in human normal liver L-02 cells. Biol Pharm Bull 31: 592–597. 18379047 10.1248/bpb.31.592

[15] Fu Q, Huang X, Shu B, Xue M, Zhang P, et al (2011) Inhibition of mitochondrial respiratory chain is involved in triptolide-induced liver injury. Fitoterapia 82: 1241–1248. 21907767 10.1016/j.fitote.2011.08.019

[16] Lee JM, Li J, Johnson DA, Stein TD, Kraft AD, et al (2005) Nrf2, a multi-organ protector? FASEB J 19: 1061–1066. 15985529 10.1096/fj.04-2591hyp

[17] Kobayashi M, Yamamoto M (2005) Molecular mechanisms activating the Nrf2-Keap1 pathway of antioxidant gene regulation. Antioxid Redox Signal 7: 385–394. 15706085 10.1089/ars.2005.7.385

[18] Li W, Kong AN (2009) Molecular mechanisms of Nrf2-mediated antioxidant response. Mol Carcinog 48: 91–104. 18618599 10.1002/mc.20465 PMC2631094

[19] Kang KW, Lee SJ, Kim SG (2005) Molecular mechanism of nrf2 activation by oxidative stress. Antioxid Redox Signal 7: 1664–1673. 16356128 10.1089/ars.2005.7.1664

[20] Li J, Jin J, Li M, Guan CW, Wang WW, et al (2012) Role of Nrf2 in protection against triptolide-induced toxicity in rat kidney cells. Toxicol lett 213: 194–202. 22820427 10.1016/j.toxlet.2012.07.008

[21] Tan KP, Kosuge K, Yang M, Ito S (2008) NRF2 as a determinant of cellular resistance in retinoic acid cytotoxicity. Free Radic Biol Med 45: 1663–1673. 18845239 10.1016/j.freeradbiomed.2008.09.010

[22] Baird L, Dinkova-Kostova AT (2011) The cytoprotective role of the Keap1-Nrf2 pathway. Arch Toxicol 85: 241–272. 21365312 10.1007/s00204-011-0674-5

[23] Curtis DK, Scott AR (2010) Nrf2 the rescue: Effects of the antioxidative/electrophilic response on the liver. Toxicol Appl Pharmacol 244: 57–65. 20122946 10.1016/j.taap.2010.01.013 PMC2860427

[24] Guerrero-Beltran CE, Calderon-Oliver M, Pedraza-Chaverri J, Chirino YI (2012) Protective effect of sulforaphane against oxidative stress: recent advances. Exp Toxicol Pathol 64: 503–508. 21129940 10.1016/j.etp.2010.11.005

[25] Baek SH, Park M, Suh JH, Choi HS (2008) Protective effects of an extract of young radish (Raphanus sativus L) cultivated with sulfur (sulfur-radish extract) and of sulforaphane on carbon tetrachloride-induced hepatotoxicity. Biosci Biotechnol Biochem 72: 1176–1182. 18460814 10.1271/bbb.70545

[26] Guerrero-Beltran CE, Calderon-Oliver M, Tapia E, Medina-Campos ON, Sanchez-Gonzalez DJ (2010) Sulforaphane protects against cisplatin-induced nephrotoxicity. Toxicol lett 192: 278–285. 19913604 10.1016/j.toxlet.2009.11.007

[27] Piao CS, Gao S, Lee GH, Kim DS, Park BH, et al (2010) Sulforaphane protects ischemic injury of hearts through antioxidant pathway and mitochondrial K(ATP) channels. Pharmacol Res 61: 342–348. 19948220 10.1016/j.phrs.2009.11.009

[28] Balasubramanian T, Senthilkumar GP, Karthikeyan M, Chatterjee TK (2013) Protective Effect of Ethyl Acetate Fraction of Stereospermum Suaveolens Against Hepatic Oxidative Stress in STZ Diabetic Rats. J Tradit Complement Med 3: 175–181. 24716175 10.4103/2225-4110.114904 PMC3924987

[29] Movahedian A, Asqary S, Mansoorkhani HS, Keshvari M (2014) Hepatotoxicity effect of some Iranian medicinal herbal formulation on rats. Adv Biomed Res 3: 12. 24592365 10.4103/2277-9175.124641 PMC3928848

[30] Chae HJ, Yim JE, Kim KA, Chyun JH (2014) Hepatoprotective effects of *Rubus coreanus* miquel concentrates on liver injuries induced by carbon tetrachloride in rats. Nutr Res Pract 8: 40–45. 24611104 10.4162/nrp.2014.8.1.40 PMC3944155

[31] Choi C, Cho H, Park J, Cho C, Song Y (2003) Suppressive effects of genistein on oxidative stress and NFkappaB activation in RAW 264.7 macrophages. Biosci Biotechnol Biochem 67: 1916–1922. 14519976 10.1271/bbb.67.1916

[32] Srividhya R, Jyothilakshmi V, Arulmathi K, Senthilkumaran V, Kalaiselvi P (2008) Attenuation of senescence-induced oxidative exacerbations in aged rat brain by (−)-epigallocatechin-3-gallate. Int J Dev Neurosci 26: 217–223. 18207349 10.1016/j.ijdevneu.2007.12.003

[33] Rubio V, Zhang J, Valverde M, Rojas E, Shi ZZ (2011) Essential role of Nrf2 in protection against hydroquinone- and benzoquinone-induced cytotoxicity. Toxicol In Vitro 25: 521–529. 21059386 10.1016/j.tiv.2010.10.021

