# Supplementary figures and images for: Activation of Nrf2 Protects against Triptolide-Induced Hepatotoxicity
Source: PLoS One. 2014 Jul 2;9(7):e100685. doi: 10.1371/journal.pone.0100685 (PMC4079517; doi:10.1371/journal.pone.0100685)

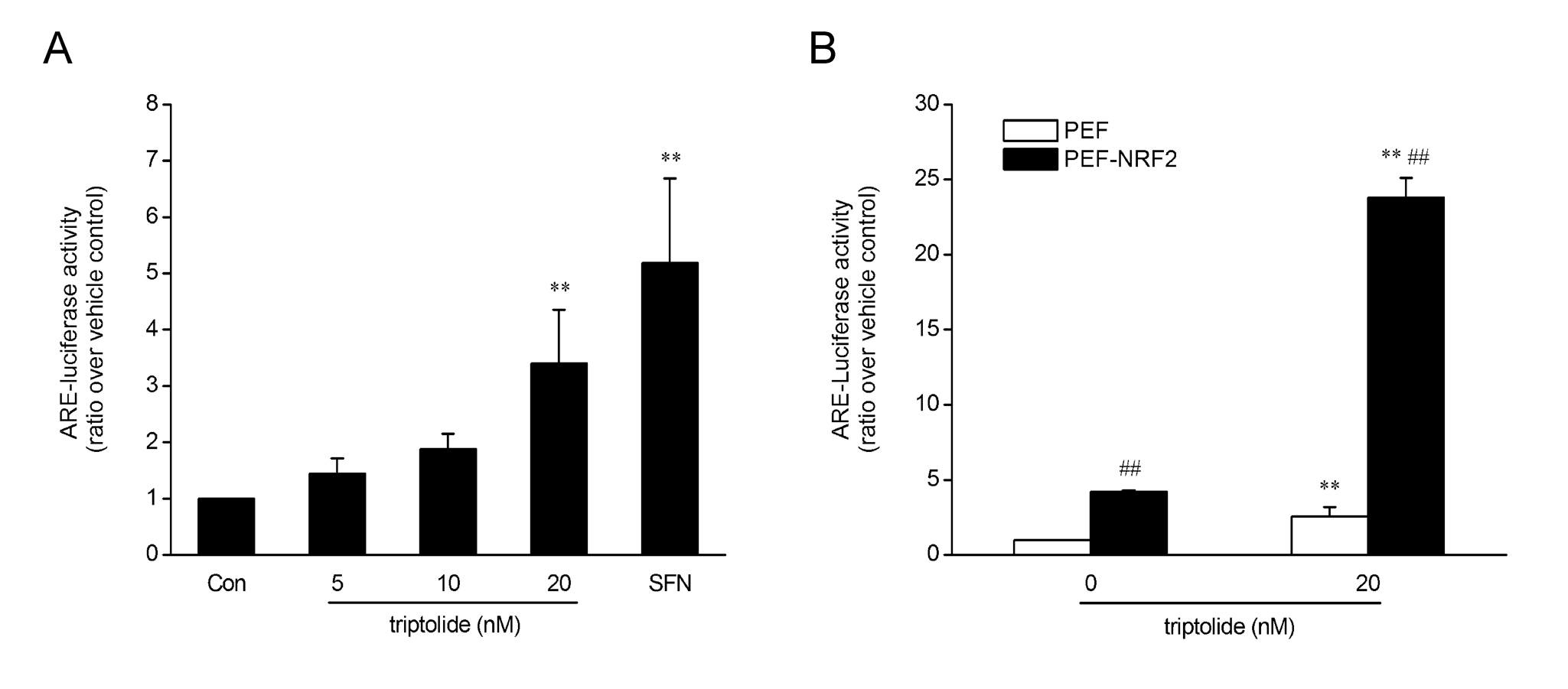

Supplement: Figure S1 — Effects of triptolide on ARE-dependent transactivation by Nrf2. (A) HepG2 cells were transfected with a pGL3 plasmid containing the ARE-motif (from the NQO1 promoter). Eighteen hours after transfection, the cells were treated with triptolide (5, 10, 20 nM) for 6 h. (B) HepG2 cells were cotransfected with a PEF empty or a Nrf2 expression vector, and a pGL3-ARE plasmid. Eighteen hours after transfection, the cells were treated with triptolide (20 nM) for 6 h. Luciferase activity was determined by calculating the firefly luciferase to renilla luciferase ratio. Data are expressed as fold of induction of luciferase activity compared to vehicle control (mean ± SD; n = 3). **P<0.01 versus vehicle control. ## P<0.01 versus cells transfected with PEF and treated with the same drug. Con: control (0.1% DMSO). (TIF) [file pone.0100685.s001.tif]

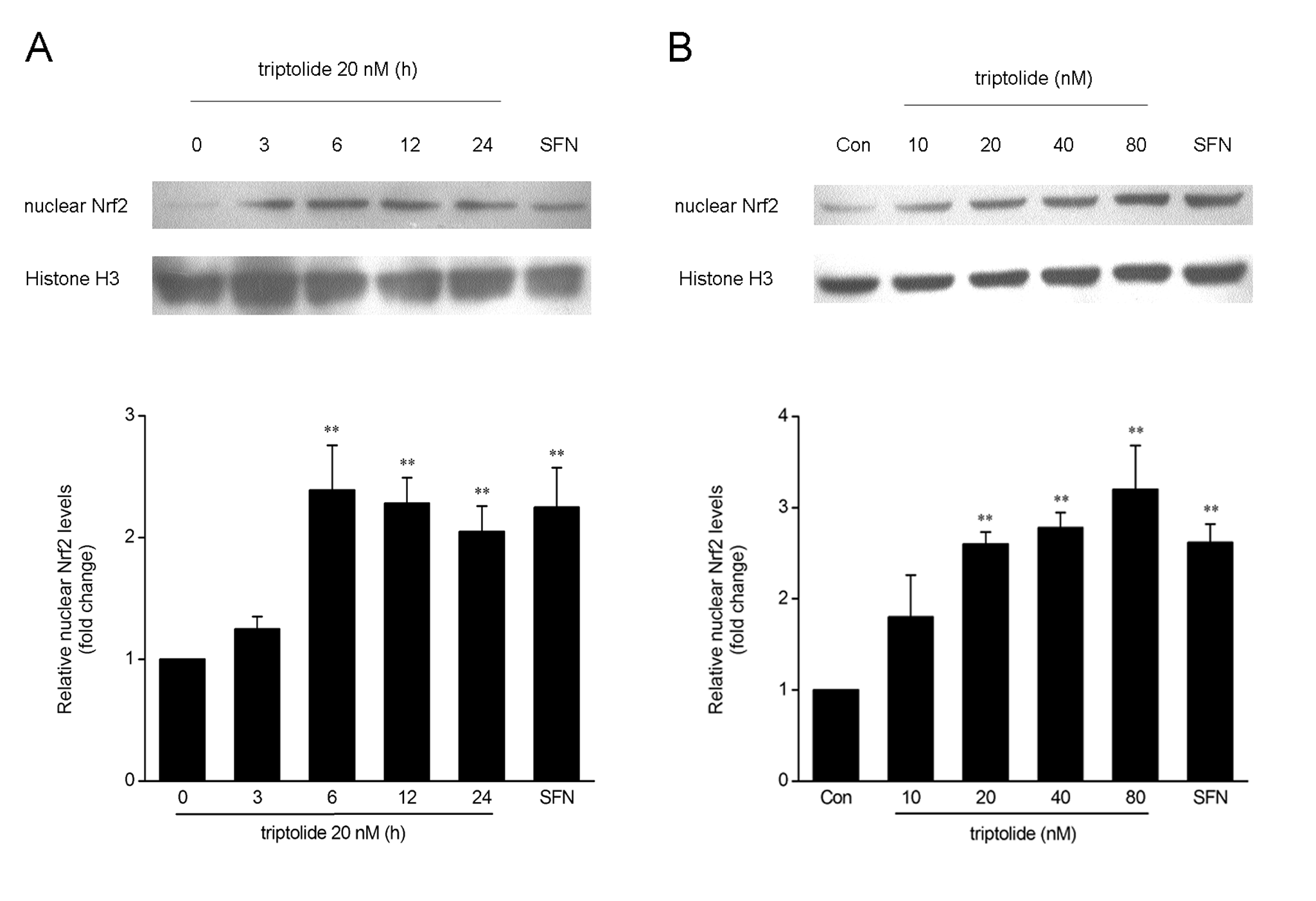

Supplement: Figure S2 — Effect of triptolide on the nuclear levels of Nrf2 protein in HepG2 cells. After being exposed to triptolide (20 nM) for the indicated time periods (A) or to the indicated concentrations of triptolide (10, 20, 40, 80 nM) for 6 h (B), the nuclear protein lysates were prepared and subjected to Western blot analysis using the indicated antibodies. The histone H3 was used as an internal control. For a positive control, HepG2 cells were treated with 5 µM sulforaphane (SFN) for 24 h (A) or 6 h (B). A representative blot from three independent experiments is shown. The density of the immunoreactive bands was analyzed, and the data are represented as the means ± SD from three independent experiments. **P<0.01 versus time 0 or vehicle control. Con: control (0.1% DMSO). (TIF) [file pone.0100685.s002.tif]

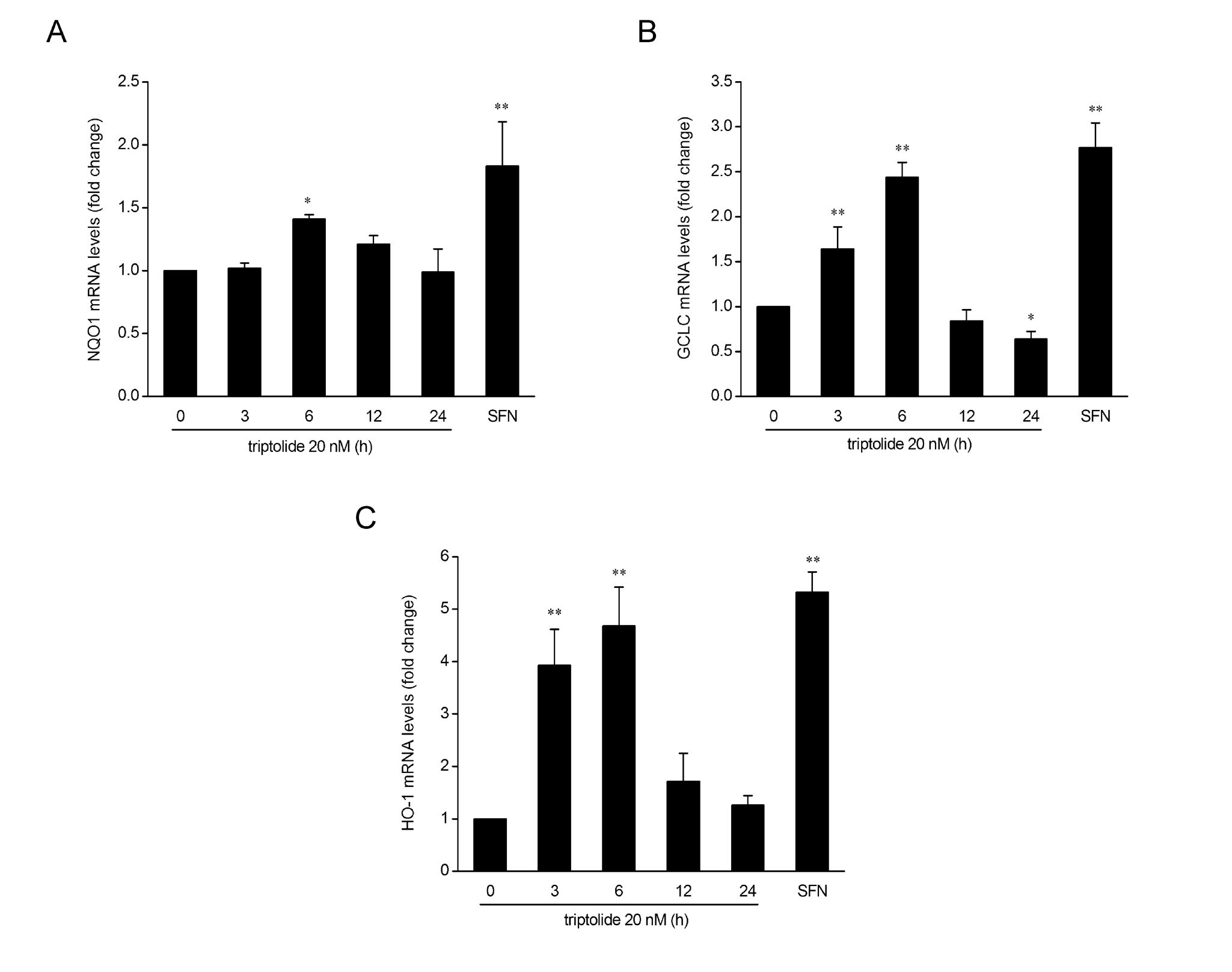

Supplement: Figure S3 — Effects of triptolide on the levels of Nrf2 target genes in HepG2 cells. HepG2 cells were treated with triptolide (20 nM) for the indicated time periods. The mRNA expressions of NQO1 (A), GCLC (B) and HO-1 (C) were analyzed by real-time PCR. β-actin was used as an internal control. For a positive control, HepG2 cells were treated with 5 µM sulforaphane (SFN) for 24 h. The data are represented as the mean ± SD from three independent experiments. *P<0.05, **P<0.01 versus time 0. (TIF) [file pone.0100685.s003.tif]
